# Amniotic membrane transplantation and conjunctival autograft combined with mitomycin C for the management of primary pterygium: A systematic review and meta-analysis

**DOI:** 10.3389/fmed.2022.981663

**Published:** 2022-11-10

**Authors:** Nada Omar Taher, Ahmed Naji Alnabihi, Reem Mahmoud Hersi, Rawan Khalid Alrajhi, Reham Ahmad Alzahrani, Waleed Talib Batais, Alaa Hesham Mofti, Saeed Abdullah Alghamdi

**Affiliations:** ^1^College of Medicine, King Saud bin Abdulaziz University for Health Sciences, Jeddah, Saudi Arabia; ^2^King Abdullah International Medical Research Center, Jeddah, Saudi Arabia; ^3^Department of Ophthalmology, Ministry of National Guard-Health Affairs, Jeddah, Saudi Arabia

**Keywords:** pterygium, autologous conjunctival transplantation, conjunctival autograft, mitomycin C, amniotic membrane transplant

## Abstract

**Background:**

Pterygium is a common ocular surface disease. Recurrence is the greatest concern in the treatment of pterygium. Thus, a standardized and effective treatment modality with minimal risk for complications is needed for the management of pterygium. The aim of this systematic review and meta-analysis was to evaluate different tissue grafting options, including conjunctival autograft (CAG) with mitomycin C (MMC), CAG alone, and amniotic membrane transplantation (AMT), for the management of primary pterygium.

**Methods:**

We searched the MEDLINE, EMBASE, and Cochrane Central Register of Controlled Trials databases for relevant studies. We included randomized controlled trials (RCTs) in which CAG + MMC and AMT were compared with surgical excision with CAG alone for the treatment of primary pterygium. The rates of recurrence and adverse events reported in the studies were also evaluated. Risk ratio (RR) was used to represent dichotomous outcomes. The data were pooled using the inverse variance weighting method. The quality of the evidence derived from the analysis was assessed using the Grading of Recommendations Assessment, Development, and Evaluation (GRADE) approach. Risk of bias was assessed using the revised Cochrane risk-of-bias tool for randomized trials.

**Results:**

Twelve RCTs (*n* = 1144) were deemed eligible and included for analysis. Five RCTs had a low risk of bias, five had some concerns, and two had a high risk of bias. Subgroup analysis showed a statistically significant reduction in the rate of pterygium recurrence after CAG + MMC (RR = 0.12; 95% confidence interval [CI], 0.02–0.63). This outcome was rated as high-quality evidence according to the GRADE criteria. There were insignificant differences between the rates of recurrence after AMT and CAG (RR = 1.51; 95% CI, 0.63–3.65). However, this result was rated as low-quality evidence. Regarding adverse events, patients treated using AMT showed significantly lower rates of adverse events than those treated using CAG (RR = 0.46; 95% CI, 0.22–0.95). However, this finding was rated as low-quality evidence as well. CAG + MMC showed a safety profile comparable to that of surgical excision with CAG alone (RR = 1.81; 95% CI, 0.40–8.31). This result was also rated as low-quality evidence.

**Conclusion:**

A single intraoperative topical application of 0.02% MMC during excision of pterygium followed by CAG has significantly shown to decrease the rate of pterygium recurrence to 1.4% with no severe complications.

## Introduction

Pterygium is an uncontrolled overgrowth of fibrovascular tissue that extends across the limbus and invades the cornea, leading to astigmatism and recurrent inflammation (1). It is a common ocular surface disease with well-documented risk factors; however, its pathogenesis is unclear, with ultraviolet exposure being identified as the main causative factor (2). Exposure to dusty, sandy, or windblown environments is also one of the main factors that contribute to the development and progression of pterygium (3, 4). A meta-analysis of 20 studies indicated that the estimated pooled prevalence of pterygium is 10.2% (5). However, its prevalence is up to 53% in some regions, such as China (5–7).

When pterygium causes obvious disfigurement and impacts vision, thereby reducing a patients’ quality of life, ophthalmologists intervene even before the threshold for surgery is reached (8, 9). Over the past few decades, the standard treatment for pterygium was bare scleral excision; however, it is associated with an unacceptably high incidence of recurrence, which can be as high as 88% in some populations (1, 10). Tissue grafting with conjunctival autograft (CAG) and amniotic membrane transplantation (AMT) has replaced bare scleral excision and become the new standard of care for pterygium owing to their relatively low recurrence rates compared to bare scleral excision (11). The risk of recurrence in patients treated using CAG ranges from 2 to 39%, that in patients treated using CAG combined with mitomycin C (MMC) ranges from 2 to 9%, and that in patients treated using AMT ranges from 3.8 to 40.9% (12).

The gold standard for pterygium removal is surgical excision with CAG (13). Several adjunctive treatment options have been developed to reduce the risk of pterygium recurrence (11). The safest and most commonly used one is MMC. MMC is an antineoplastic antibiotic that selectively inhibits the synthesis of DNA, RNA, and protein in all cells. MMC interferes with cell proliferation, making it a good option for controlling endothelial cell proliferation during pathophysiological angiogenesis (9, 14). However, the exact efficacy and safety of MMC is unclear.

The results of previous studies on the pathophysiology and management of pterygium do not clarify some unclear aspects of this common ocular surface disease. For instance, no previous systematic review collectively described the roles of different tissue grafting options as individual treatments or in combination with adjunctive therapies for the treatment of pterygium (1, 3, 15). Since recurrence is the greatest concern in the treatment of pterygium, a standardized and effective treatment with a very low risk of complications is needed, especially considering that repeated surgical procedures often worsen the disease (16). Therefore, the aim of this systematic review and meta-analysis was to comprehensively evaluate the efficacy and safety of CAG combined with MMC and AMT with or without MMC compared to surgical excision with CAG alone for the treatment of primary pterygium.

## Methods

This systematic review was conducted according to a pre-specified protocol registered in PROSPERO (CRD42022297725) and the Preferred Reporting Items for Systematic Reviews and Meta-Analysis (PRISMA) checklist (17).

### Search strategy

We systematically searched the MEDLINE, EMBASE, and Cochrane Central Register of Controlled Trials databases for relevant articles published from the dates of the establishment of the databases to January 10, 2022. No date or language restrictions were applied during the search. The complete search strategy is outlined in the Supplementary material. We manually searched the references of the retrieved articles for potentially relevant randomized controlled trials (RCTs) that were not found during the systematic search.

### Eligibility criteria

Studies that included participants who underwent surgical excision of primary pterygium were included in this systematic review and meta-analysis. The interventions applied in the included studies were surgical excision with CAG alone, CAG + MMC, or AMT with or without MMC. The evaluated outcomes were recurrence rates and adverse events. Trials in which patients with recurrent pterygium or any ocular surface lesions other than pterygium were the study population and those in which the participants were treated using adjunctive therapies other than MMC were excluded from this systematic review and meta-analysis.

Mitomycin C was the only adjunctive therapeutic option investigated in the present study. This is because most of the other adjuvant options have been abandoned for MMC owing to its relative superiority in terms of safety (9). Patients who were treated using surgical excision with CAG were the control group in the present study because most cornea specialists consider surgical excision with CAG the gold standard for the treatment of primary pterygium (16).

### Study selection and data extraction

Two reviewers independently and jointly screened the titles and abstracts of the extracted articles according to the eligibility criteria. The full texts of potentially eligible articles were assessed and the data of the eligible studies were extracted for analysis. Discrepancies were resolved through consensus or discussion with a third reviewer before the analyses.

### Risk of bias assessment

Two reviewers independently and jointly assessed the risks of bias in the eligible RCTs using the revised Cochrane risk-of-bias tool (18). Each study was reviewed and its risk of bias was categorized as follows: “high risk,” “low risk,” or “some concerns.” Discrepancies between the reviewers were resolved through discussion until agreement was reached. We assessed each outcome’s potential for publication bias through visual inspection of a funnel plot with risk ratios (RR) and standard errors. Publication bias was considered to be possible if the funnel plot was asymmetrical.

### Meta-analysis

Data analysis was performed using the RevMan software version 5.3 (Cochrane Collaboration). All statistical analyses were performed using a random-effects model. We adopted a 95% confidence level and threshold of *P* < 0.05. Statistical heterogeneity was assessed using *I*^2^ and the *P*-values derived from the chi-square test. Dichotomous outcomes (recurrence rate and adverse events) were expressed as RRs and pooled using the inverse variance weighting method. We performed a subgroup analysis based on the types of interventions (CAG + MMC and AMT subgroups). Although subgroup analyses at multiple follow-up timespoints would have provided an excellent representation of the efficacy and safety of the interventions, such data were not reported in most of the included RCTs. The quality of evidence for each outcome was assessed using the Grading of Recommendations Assessment, Development, and Evaluation (GRADE) criteria.

## Results

**FIGURE 1:**
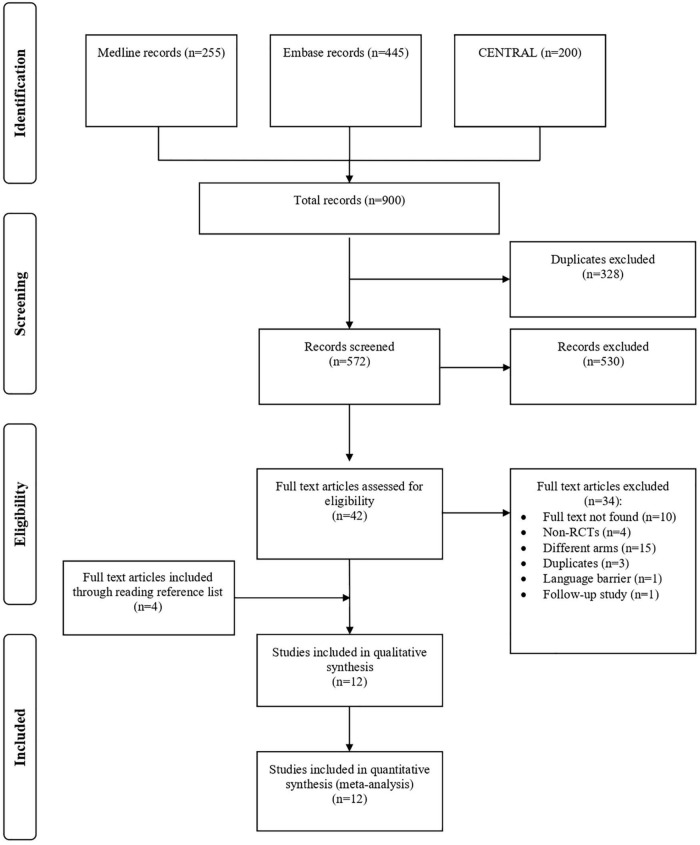
Study flow diagram.

Figure 1 shows the flowchart of the inclusion process and exclusion criteria of this study. A total of 900 articles were retrieved during the literature search. After screening the articles, 328 duplicates were identified and excluded. The titles and abstracts of the remaining articles were read and 42 potentially eligible studies were assessed for inclusion. Ultimately, 12 RCTs were deemed eligible and included in the meta-analysis. Regarding the interventions, CAG + MMC was evaluated in three RCTs, whereas AMT was evaluated in nine RCTs. AMT + MMC was not evaluated in any of the studies.

### Characteristics of the included trials

A total of 1,144 participants were enrolled in the 12 RCTs. The participants were randomly assigned to a treatment group. A total of 557 participants received CAG alone, 520 received AMT, and 67 received CAG + MMC. The mean age of the patients ranged from 42 to 63 years for the CAG arm, 37–62 years for the AMT arm, and 41–48 years for the CAG + MMC arm. Recurrence of pterygium six months after surgery was reported in seven RCTs, recurrence 12 months after surgery was reported in four RCTs, and recurrence 48 months after surgery was reported in one RCT. The most commonly used sutures were 8-0 vicryl and 10-0 nylon. The detailed characteristics of the included studies are presented in Table 1.

**TABLE 1:** Characteristics of the included studies.

References	Interven-tion	MMC specifics (dose, timing, etc.)	Number of participants		Number of eyes		Age (years)*		Gender		Type of sutures used	Planned follow-up (months)	The definition of pterygium recurrence
									
			CAG	Interven-tion	CAG	Interven-tion	CAG	Interven-tion	Male	Female			
Frucht-Pery et al. (19)	CAG vs. combined CAG with MMC	Intraoperative application of MMC 0.02% for 1 min (before the transplantation procedure was performed)	30	30	NR	NR	43.4 ± 12.2	41.8 ± 11.8	NR	NR	10-0 nylon stitches	13 months	Vascular invasion through the limbal area into the clear cornea.
Prajna et al. (23)	CAG vs. AMT	–	33	33	33	33	Range = 44–64	Range = 44–64	11	22	8- 0 vicryl suture for the conjunctival side of the transplant; 10- 0 nylon suture for the limbal side	12 months	The presence of additional fibrous tissue in the excised area that did not invade the cornea or fibrovascular tissue invasion through the cornea.
Agahan et al. (20)	CAG vs. combined CAG with MMC	Application of intraoperative 0.02% MMC in the bare sclera and head of the pterygium for 3 min and then washed off with normal saline solution after excision of the pterygium	NR	NR	15	17	44.73 ± 11.99	44.88 ± 3.29	CAG = 6, CAG + MMC = 8	CAG = 9, CAG + MMC = 9	10-0 nylon stitches	6 months	Fibrovascular proliferation encroaching onto the cornea coming from the original pterygium site.
Balakrishna et al. (24)	CAG vs. AMT		45	45	NR	NR	46.53 ± 13.51	37.89 ± 10.85	CAG = 19, AMT = 31	CAG = 26, AMT = 14	NR	6 months	Fibrovascular growth beyond the limbus onto the cornea.
Dos Santos Martins et al. (21)	CAG vs. combined CAG with MMC	0.1 ml of 0.02% MMC subconjunctival injection (1 month vs. 2 weeks pre-op)	29	1 month pre-op = 16 2 weeks pre-op = 20	29	1 month pre-op = 16 2 weeks pre-op = 20	Range = 21–84	Range = 21–84	31	34	Nylon 10-0	24 months	Fibrovascular growth beyond the limbus onto the cornea.
Pan et al. (25)	Hyperdry AMT vs. CAG	–	59	71	62	79	63.05 ± 6.678	62.32 ± 7.030	AMT = 31, CAG = 26	AMT = 40, CAG = 33	10-0 nylon	12 months	Fibrovascular growth beyond the limbus onto the cornea.
Ma et al. (26)	CAG vs. AMT	–	50	71	56	80	56.4 ± 11.9	56.7 ± 11.3	CAG = 19, AMT = 35	CAG = 31, AMT = 36	8-0 vicryl	6 months	Fibrovascular growth beyond the limbus onto the cornea.
Luanratanakor et al. (27)	CAG vs. AMT	–	106	148	106	148	44.75 ± 11.44	45.31 ± 12.84	CAG = 40, AMT = 52	CAG = 66, AMT = 96	Interrupted 10-0 nylon sutures	6 months	Vascular invasion through the limbal area into the clear cornea.
Tananuvat and Martin. (30)	CAG vs. AMT	–	41	39	42	44	44.81 ± 8.77	41.93 ± 9.07	CAG = 18, AMT = 16	CAG = 23, AMT = 23	8-0 vicryl	6 months	Post-operative regrowth of fibrovascular tissue crossing the limbus onto the clear cornea in the area of previous pterygium excision.
Toker and Eraslan. (28)	CAG vs. AMT	–	40	34	43	39	52 ± 13.7	49.8 ± 14.1	CAG = 18, AMT = 16	CAG = 16, AMT = 15	NR	12 months	Conjunctival fibrovascular extension to the limbus (limbal recurrence) or more than 1 mm onto the cornea.
Liang et al. (29)	CAG vs. AMT	–	NR	NR	81	52	Range = 32–85	Range = 30–81	CAG = 32, AMT = 20	CAG = 49, AMT = 32	10-0 nylon stitches	12 months	The presence of conjunctival hyperemia, neovascularization, and pterygium tissue invasion.*

CAG, conjunctival autograft; AMT, amniotic membrane transplantation; MMC, mitomycin C; Pre-op, pre-operative; NR, not reported.

*Age of participants represented as mean ± standard deviation; when unavailable, it was represented as range.

### Risk of bias assessment

**FIGURE 2:**
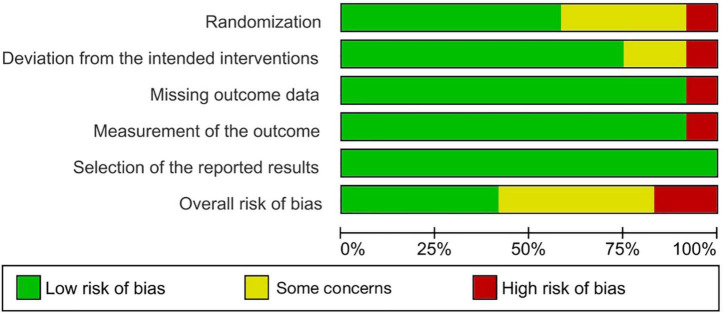
Risk of bias graph.

**FIGURE 3:**
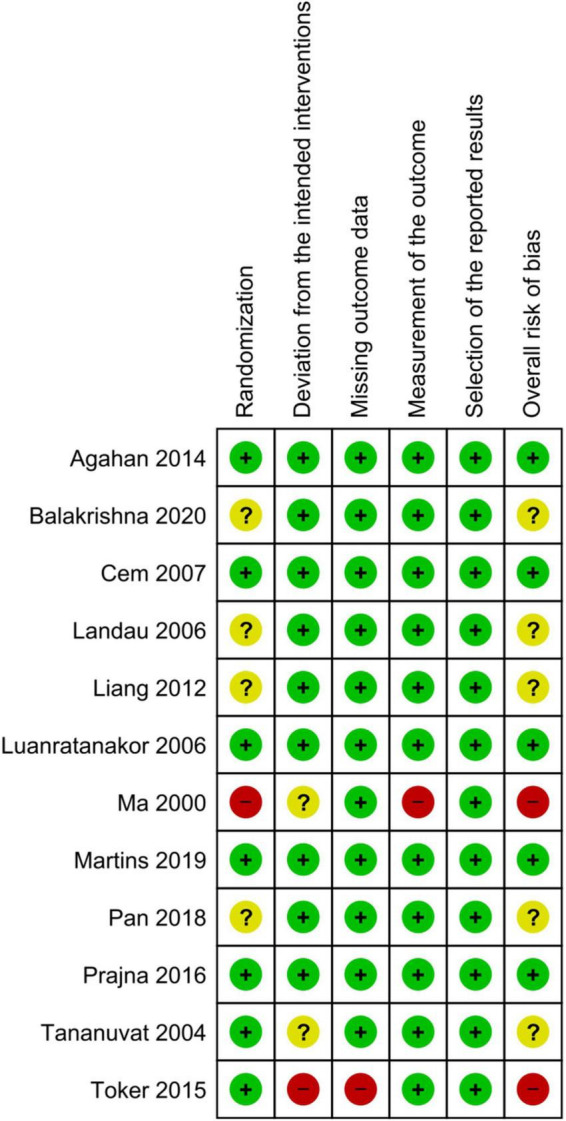
Risk of bias summary.

Five of the 12 RCTs had a low risk of bias, five had some concerns, and two had a high risk of bias. Figures 2, 3 show the assessment of the risk of bias in all the included RCTs.

### Recurrence rate

**FIGURE 4:**
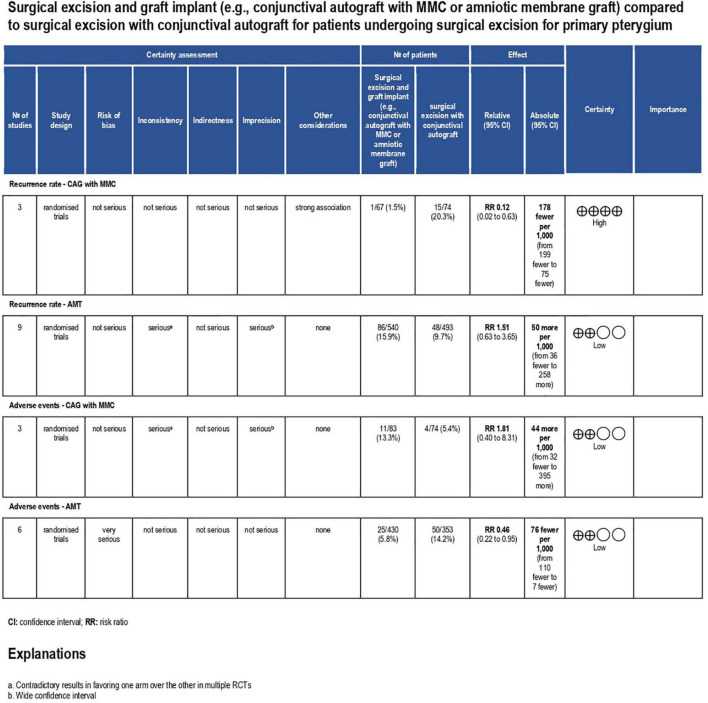
Grading of recommendations assessment, development and evaluation (GRADE) evidence profile. CI, confidence interval; RCT, randomized controlled trial; ROP, retinopathy of prematurity; RR, risk ratio; CAG, conjunctival autograft; AMT, amniotic membrane transplantation; MMC, mitomycin C.

**FIGURE 5:**
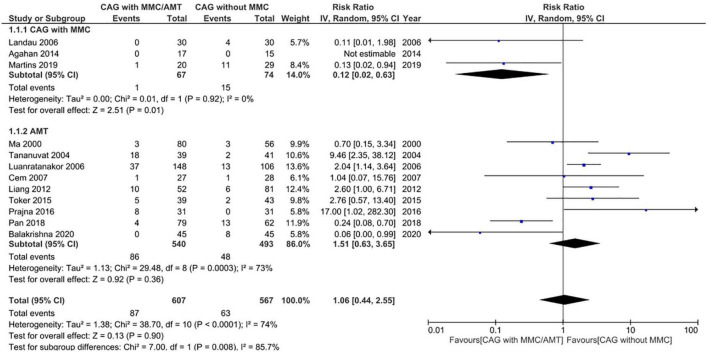
Forrest plot of rate of recurrence. CI, confidence interval; IV, inverse variance; CAG, conjunctival autograft; AMT, amniotic membrane transplantation; MMC, mitomycin C.

**FIGURE 6:**
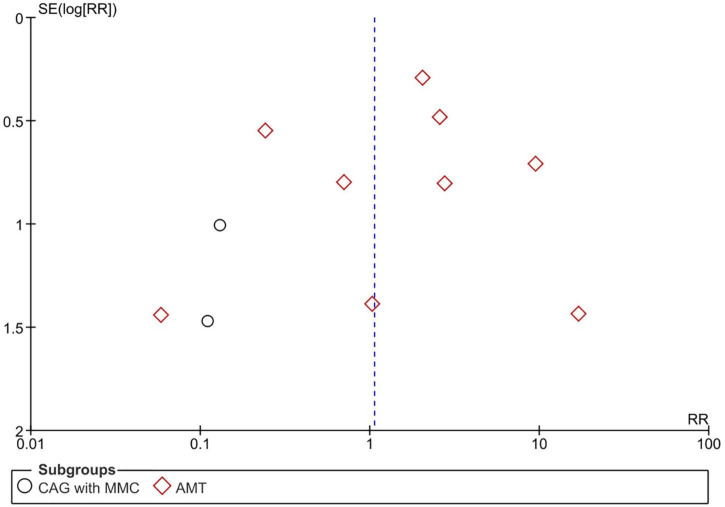
Funnel plot of rate of recurrence. SE, standard error; RR, risk ratio; CAG, conjunctival autograft; AMT, amniotic membrane transplantation; MMC, mitomycin C.

Pterygium recurrence was reported in all 12 RCTs (*n* = 1,144 participants) (19–30). The CAG + MMC intervention was associated with significant reduction in recurrence rates compared to CAG alone (RR = 0.12; 95% confidence interval [CI], 0.02–0.63; *P* < 0.05; *I*^2^ = 0%). The rates of recurrence after CAG with and without MMC were 1.4 and 11.3%, respectively. The result regarding the rate of recurrence after CAG + MMC was rated as high-quality evidence according to the GRADE certainty of evidence criteria (Figure 4). However, there were no statistically significant differences between AMT and CAG in terms of recurrence rate. The rate of pterygium recurrence after AMT was 16.5% (RR = 1.51; 95% CI, 0.63–3.65; *P* = 0.36; *I*^2^ = 73%) (Figure 5). This finding was rated as low-quality evidence (Figure 4). The funnel plot was symmetric; therefore, publication bias was unlikely (Figure 6).

### Adverse events

**FIGURE 7:**
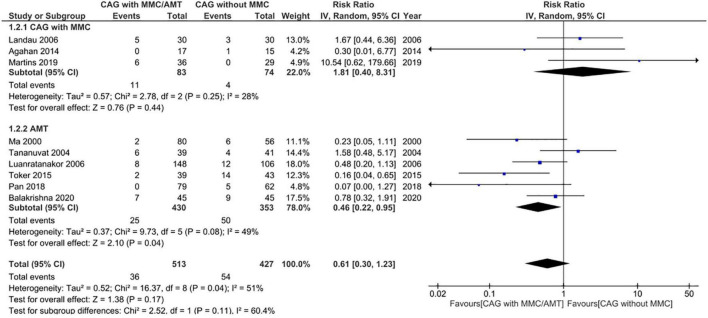
Forrest plot of adverse events. CI, confidence interval; IV, inverse variance; CAG, conjunctival autograft; AMT, amniotic membrane transplantation; MMC, mitomycin C.

**FIGURE 8:**
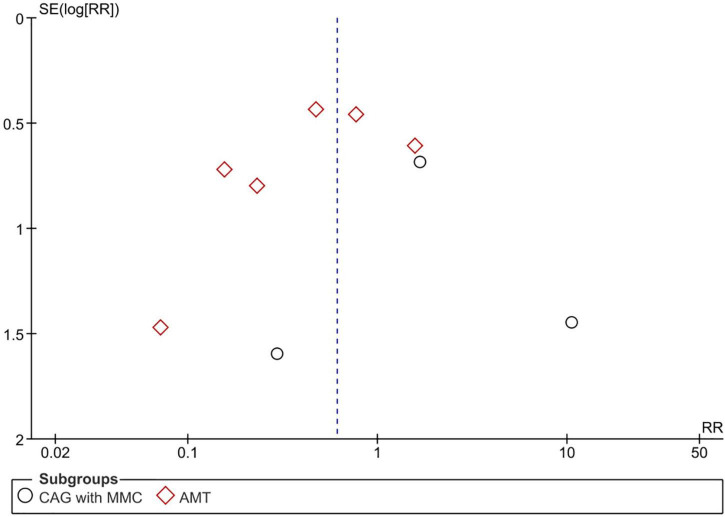
Funnel plot of adverse events. SE, standard error; RR, risk ratio; CAG, conjunctival autograft; AMT, amniotic membrane transplantation; MMC, mitomycin C.

Adverse events were reported in nine RCTs (*n* = 890) (19–21, 24–28, 30). Patients treated using CAG + MMC and CAG alone showed similar rates of adverse events (RR = 1.81; 95% CI, 0.40–8.31; *P* = 0.44; *I*^2^ = 28%); however, the range of the CI was wide. This could be attributed to the relatively small sample size of this subgroup. Patients treated using AMT showed significantly lower adverse event rates than those treated using CAG (RR = 0.46; 95% CI, 0.22–0.95; *P* < 0.05; *I*^2^ = 49%) (Figure 7). The funnel plot was symmetric; therefore, publication bias was unlikely (Figure 8). However, the quality of the evidence regarding adverse events in both subgroups was low (Figure 4).

## Discussion

In this systematic review and meta-analysis of 12 RCTs with a total of 1,144 participants, we compared the efficacy and safety of surgical excision with AMT or CAG + MMC with those of surgical excision with CAG alone for the treatment of primary pterygium. Subgroup analysis showed a statistically significant reduction in recurrence rates after treatment using CAG + MMC. Regarding adverse events, patients treated using AMT showed significantly lower rates of adverse events than those treated using CAG.

In the most recent Cochrane systematic review, CAG was compared with AMT for the treatment of both primary and recurrent pterygia. In that review, the risk of recurrence 6 months after surgery was significantly lower in the CAG group than in the AMT group. In addition, there was no clinically or statistically significant difference between the CAG and AMT groups in terms of recurrence in patients with primary pterygium 3 months after surgery (1, 31). This could be explained by the fact that most recurrence events occur 6 months after surgery rather than 3 months after (32). However, it should be noted that subgroup analysis of recurrence in patients with primary pterygium 6 months after surgery has not been conducted in any study to date.

The results of the present study demonstrate the superiority of CAG + MMC over other tissue grafting techniques for the treatment of pterygium. This finding is consistent with those of a network meta-analysis of 2,483 patients in which the efficacy of different adjuvants for the prevention of recurrence following pterygium surgery were compared. In that meta-analysis, the MMC + CAG group showed lower rates of recurrence compared to the CAG alone group (15, 33). In another study of 75 patients, most of whom had advanced primary pterygium, the use of 0.02% MMC (the same dose used in the RCTs included in the present study) was associated with lower rates of recurrence than 0.01% MMC; however, the result was not statistically significant (33). The outcomes of intraoperative and post-operative administration of MMC have been compared in some studies and the results showed that the former is much safer than the latter (34, 35).

Most adverse events related to MMC are reported after post-operative administration (12). Post-operative topical administration of MMC is no longer recommended because of possible drug misuse (i.e., uncontrolled and prolonged use of the drug by the patients), which leads to severe ocular complications (36). Intraoperative administration of MMC is generally preferred because it is safer than post-operative daily topical administration. However, scleral melting after intraoperative administration of MMC has been reported. Maintaining the epithelium over the intact operated area is crucial for the prevention of scleral melting (37, 38). Additionally, cautious selection of candidates for the administration of MMC is essential for the prevention of severe ocular complications. In this context, MMC should not be administered to patients with abnormal ocular surfaces who have a high risk for excessive inflammation or delayed epithelialization (e.g., patients with immune disorders, blepharitis, or dry eyes) (37, 39–41).

The use of MMC has been discouraged in several studies because it causes scleral thinning (37, 38, 42). Delayed epithelialization and iritis, which may occur after both intraoperative and post-operative administration of MMC, are also severe complications associated with the use of MMC in pterygium surgery. However, no serious complication was reported in any of the RCTs included in the present study (42). Landau et al. reported that no significant adverse effects were observed in any of the patient groups in their study, including the MMC arm (12). Similarly, in the trial by Agahan et al., steroid-induced elevation of intraocular pressure and formation of Tenon’s cyst were reported; however, they were not considered related to the use of MMC (20). Martins et al. also reported no MMC-related side effects during the follow-up period in their study (21). Most of the adverse events reported in the RCTs analyzed in the present study were present in the other subgroups (i.e., the CAG and AMT subgroups). In addition, the side effects were transient and did not have any serious vision-threatening effects.

The results of the present study support the use of CAG combined with intraoperative administration of 0.02% MMC for the treatment of pterygium because it has the lowest risk of recurrence compared to the other studied interventions; in addition, it is not associated with any serious adverse effects. However, it must be noted that the optimal MMC dose is yet to be established. Our recommendations regarding the MMC dose and route are based on the results of three RCTs that were included in the CAG + MMC subgroup (9, 17, 18). Although MMC is associated with decreased rates of recurrence after pterygium excision, a conventional route of administration, careful dosing, and patient selection are recommended.

The present study is the most comprehensive systematic review and meta-analysis of published RCTs with high-quality evidence derived from a relatively large sample size to date. In addition, a subgroup analysis of the interventions was performed to improve the clinical relevance of the results. Furthermore, the GRADE criteria were applied to each studied outcome in this high-quality systematic review and meta-analysis, ensuring a transparent assessment of the certainty of the evidence and an explicit and comprehensive evaluation of the outcomes of alternative management strategies. This enabled us to provide reliable and pragmatic recommendations. To our knowledge, no other systematic review on the safety and efficacy of CAG + MMC or AMT in patients with primary pterygium involved the evaluation of outcomes using the GRADE criteria.

This review had several limitations. First, variations in MMC doses and follow-up periods across RCTs may have affected the results. Second, the studies included in this meta-analysis showed some heterogeneity, probably owing to variability in patient populations and treatment protocols. Third, the included RCTs have some risks of bias, specifically relating to randomization techniques, deviation from the intended intervention, missing outcome data, and outcome measurements. Finally, the definitions of pterygium recurrence in the RCTs were inconsistent.

## Conclusion

A single intraoperative topical administration of 0.02% MMC during excision of pterygium, followed by CAG transplantation, has shown to decrease the pterygium recurrence rate to 1.4% without any serious complications. Future studies are needed to determine the lowest effective dose of MMC that prevents pterygium recurrence without causing complications, as well as the optimal route and timing of administration. In addition, well-conducted RCTs are needed for the evaluation and comparison of the available sutureless techniques for the treatment of pterygium, which is an interesting and novel research topic.

## Data availability statement

The original contributions presented in this study are included in the article/Supplementary material, further inquiries can be directed to the corresponding author.

## Author contributions

NT, AA, and RH contributed to the study conceptualization, study design, data extraction, statistical analysis/interpretation, risk of bias assessment, and writing/editing of the final manuscript. RKA and RAA contributed to the study design, data extraction, statistical analysis and interpretation, and writing and editing of the final manuscript. WB contributed to the data extraction, statistical analysis/interpretation, risk of bias assessment, and writing/editing of the final manuscript. SA and AM contributed to the statistical analysis, interpretation, and writing and editing of the final manuscript. All authors contributed to the article and approved the submitted version.
