# Supplementary material for: Amniotic membrane transplantation and conjunctival autograft combined with mitomycin C for the management of primary pterygium: A systematic review and meta-analysis
Source: Front Med (Lausanne). 2022 Nov 10;9:981663. doi: 10.3389/fmed.2022.981663 (PMC9684179; doi:10.3389/fmed.2022.981663)
Supplement: Supplementary file 1 [file Data_Sheet_1.DOCX]

Supplementary Material

Amniotic Membrane Transplantation and Conjunctival Autograft Combined with Mitomycin C for the Management of Primary Pterygium: A Systematic Review and Meta-Analysis

Nada Omar Taher, Ahmed Naji Alnabihi, Reem Mahmoud Hersi, Rawan Khalid Alrajhi, Reham Ahmad Alzahrani, Waleed Talib Batais, Alaa Hesham Mofti, Saeed Abdullah AlGhamdi

Search strategy for Medline, Embase, and Cochrane Central Register of Controlled Trials (CENTRAL), last performed on **10/1/2022** (n=572)

1. exp pterygium/

2. pterygium$.mp.

3. exp pterygium/ or exp excision/

4. pterygium excision$.mp.

5. Amniotic membrane transplantation$.mp.

6. Amniotic membrane graft$.mp.

7. Amniotic membrane$.mp.

8. 5 or 6 or 7

9. Conjunctival flap$.mp.

10. Conjunctival autograft$.mp.

11. 9 or 10

12. exp randomized controlled trial/

13. Randomized controlled trial$.mp.

14. exp randomized controlled trial/ or exp clinical trial/

15. RCT$.mp.

16. exp "randomized controlled trial (topic)"/ or exp clinical trial/ or exp "controlled clinical trial (topic)"/ or exp randomized controlled trial/ or exp controlled clinical trial/

17. trial$.mp.

18. exp clinical trial/

19. clinical trial$.mp.

20. 12 or 13 or 14 or 15 or 16 or 17 or 18 or 19

21. 1 or 2 or 3 or 4

22. 5 or 6 or 7 or 9 or 10

23. exp amnion/

24. exp autograft/ or exp conjunctiva/

25. 22 or 23 or 24

26. exp mitomycin C/

27. 25 or 26

28. 20 and 21 and 27

29. remove duplicates from 28
